# Adjunctive role of middle meningeal artery embolization in patients with surgical treatment of unilateral chronic subdural hematoma: a systematic review and meta-analysis of randomized controlled trials

**DOI:** 10.3389/fsurg.2025.1623619

**Published:** 2025-07-25

**Authors:** Johannes Wach, Martin Vychopen, Erdem Güresir

**Affiliations:** Department of Neurosurgery, University Hospital Leipzig, Leipzig, Germany

**Keywords:** chronic subdural hematoma, meta-analysis, middle meningeal artery embolization, reoperation, surgical evacuation, unilateral

## Abstract

**Background:**

Chronic subdural hematoma (cSDH) is a common neurological condition, with high recurrence rates after surgical evacuation, posing significant challenges for patient outcomes. Middle meningeal artery embolization (MMAE) has emerged as a potential adjunctive therapy to reduce recurrence and reoperation rates. This meta-analysis evaluates the impact of MMAE on recurrence and reoperation rates in surgically treated unilateral subdural hematoma patients.

**Methods:**

A systematic review and meta-analysis were conducted, adhering to PRISMA guidelines. Randomized controlled trials comparing surgical evacuation with and without adjunctive MMAE were included. The primary outcomes were recurrence and reoperation rates within 90 days. Pooled odds ratios (ORs) were calculated using a random-effects model. Statistical heterogeneity was assessed using the I^2^ statistic.

**Results:**

Two trials involving 965 patients met inclusion criteria. 478 patients underwent surgery with MMAE, and 487 patients underwent only surgery. MMAE reduced reoperation rates from 6.0% in controls to 2.5% in the MMAE group (OR: 0.41, 95% CI: 0.20–0.82; *p* = 0.01), with no significant heterogeneity (I^2^ = 0%). Recurrence rates were lower in the MMAE group (5.2% vs. 9.2%, OR: 0.52, 95% CI: 0.17–1.59; *p* = 0.25), but the difference was not statistically significant.

**Conclusion:**

MMAE significantly reduces the risk of reoperation in surgically treated unilateral subdural hematoma patients and may also reduce recurrence rates. These findings support the integration of MMAE as an adjunct to surgery in selected patients.

## Graphical Abstract

**Figure:**
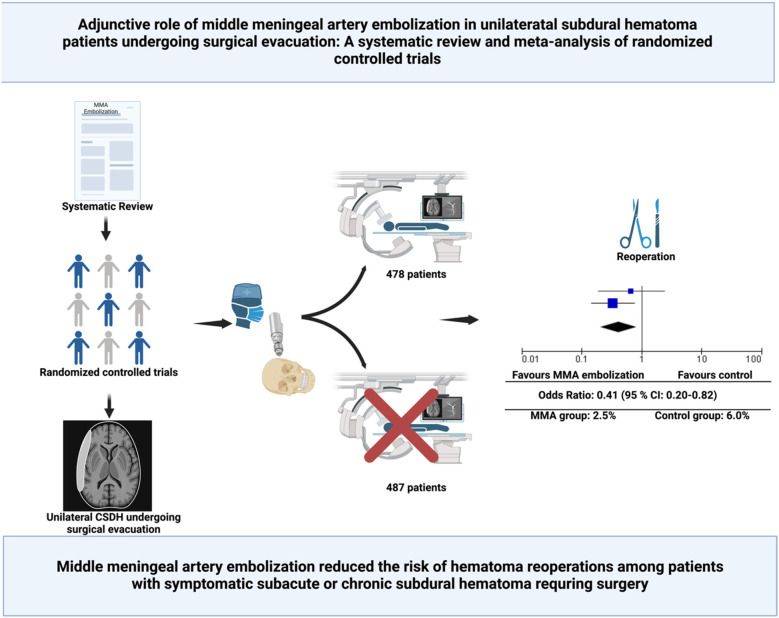

## 1 Introduction

Chronic subdural hematoma (cSDH) is one of the most prevalent neurological conditions encountered in neurosurgical practice, especially in the aging population, with its incidence projected to rise globally due to increasing longevity and the widespread use of anticoagulants and antiplatelet therapies (1, 2). This condition is frequently precipitated by minor head trauma, initiating a cascade of inflammation and vascular proliferation that results in the formation of fragile subdural membranes prone to recurrent microhemorrhages (3). Such pathophysiology often leads to persistent or recurrent hematomas, despite effective initial surgical treatment with burr hole drainage (4).

Recurrence rates after surgery remain high, ranging from 8% to 20%, posing a significant challenge in terms of patient outcomes and healthcare resource utilization (5–7). Alternative and adjunctive strategies have emerged, including anti-inflammatory drug therapies, corticosteroids, and novel endovascular approaches like middle meningeal artery embolization (MMAE) (2, 8, 9). While corticosteroids show promise in modulating the underlying inflammatory response, their additive use to surgery has been associated with mixed outcomes, particularly in reducing recurrence rates while increasing adverse events and impairing functional outcome (7).

Among these strategies, MMAE has garnered significant interest as an approach aiming to disrupt the vascular supply to the pathological subdural membranes (9). This endovascular technique, when combined with surgical evacuation or in conservative patients as a stand-alone treatment, has been suggested in recent randomized controlled trials to significantly reduce recurrence rates, reoperation needs, and associated morbidity (10–14). However, these trials address the role of MMAE in different settings (e.g., stand-alone, combined with surgery, unilateral or bilateral SDH). Against this backdrop, there is the need for a pooled and synthetized data in a meta-analysis of unilateral subacute or chronic subdural patients undergoing surgical evacuation with or without MMAE to investigate the additive impact of MMAE in a homogeneous cohort which represents the main clinical cohort.

This systematic review and meta-analysis aim to synthesize evidence from randomized controlled trials to elucidate the adjunctive role of MMAE in the surgical management of unilateral cSDH, focusing on recurrence and reoperation. By integrating findings from recent high-quality studies, we seek to provide a comprehensive evaluation of MMAE's potential to refine the surgical management paradigm for unilateral cSDH.

## 2 Methods

### 2.1 Study design

This meta-analysis was conducted following the Preferred Reporting Items for Systematic Reviews and Meta-Analyses (PRISMA) guidelines (PRISMA checklist in Supplementary Figure S1) and the Cochrane Handbook for Systematic Reviews of Interventions (Version 6.5) (15).

### 2.2 Eligibility criteria

The inclusion criteria were formulated using the PICOS framework (16). Population: Adult patients with unilateral subacute or chronic subdural hematoma undergoing surgical evacuation. Intervention: Adjunctive middle meningeal artery embolization (MMAE). Comparator: Surgical evacuation without adjunctive MMAE. Outcomes: Primary outcomes included recurrence or reoperation rates at predefined time points (90-days). Study Design: Randomized controlled trials (RCTs) with sufficient reporting of outcomes stratified by surgical status.

Studies were excluded if they involved bilateral subdural hematomas, focused on conservative management without surgery, or provided insufficient data for meta-analysis.

### 2.3 Definition of endpoints

Recurrence is defined as the reappearance or progression of subdural hematoma (SDH), confirmed by imaging or clinical symptoms, within 90 days of the index treatment. This includes an increase in hematoma thickness exceeding 10 mm, or an increase of more than 3 mm compared to baseline, with or without associated neurological deterioration. Reoperation refers to any repeat surgical intervention performed within 90 days to manage hematoma recurrence or progression, as determined by clinical or imaging criteria.

### 2.4 Literature search

A comprehensive literature search was conducted using PubMed, the Cochrane Library, and Google Scholar. The search covered all articles published from database inception to November 2024. Search terms included a combination of keywords and MeSH terms for chronic subdural hematoma, MMAE, and surgical treatment. The detailed search strategy is provided in Supplementary Table 1.

### 2.5 Study selection

**Figure 1:**
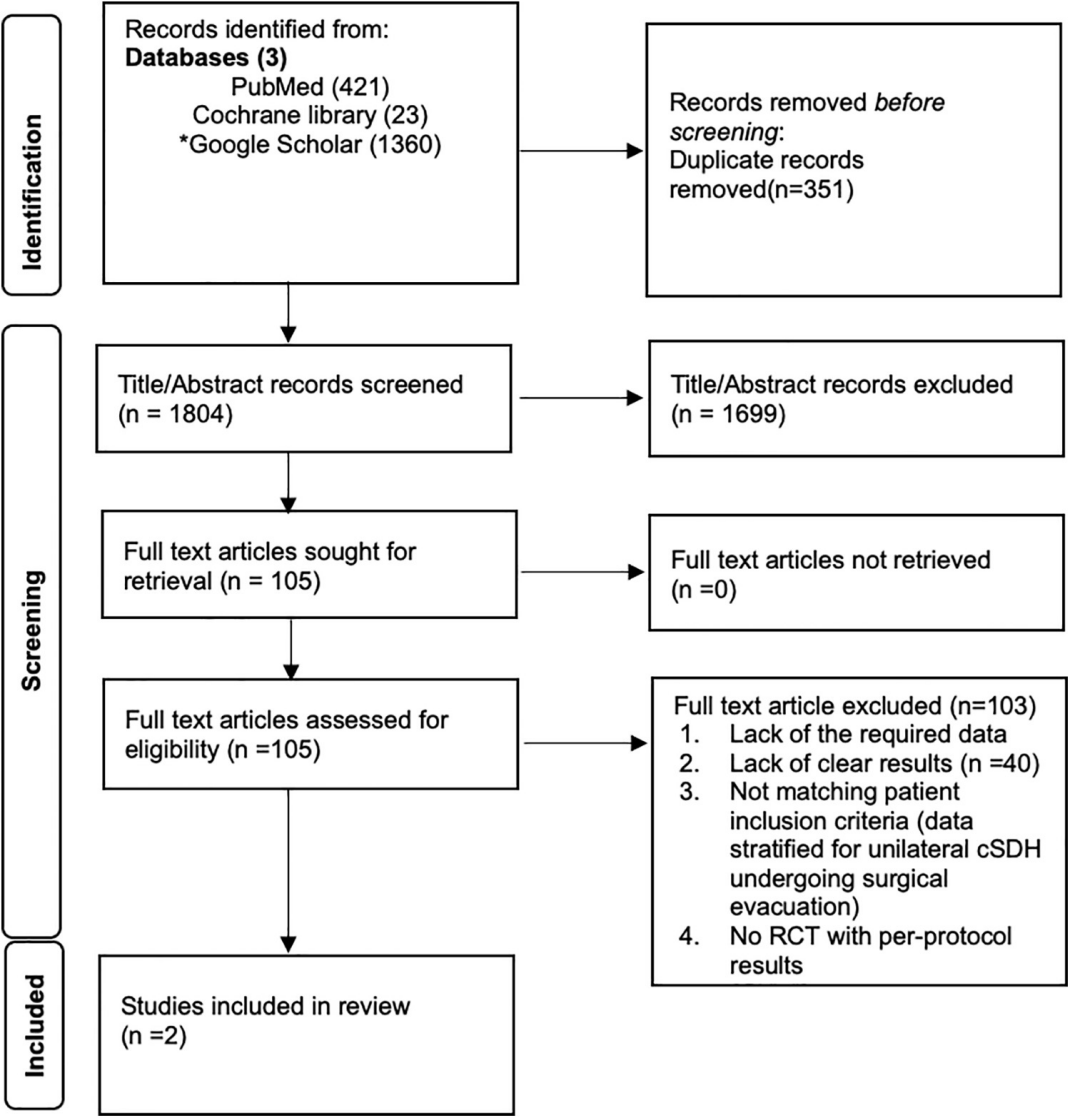
PRISMA flowchart for study selection.

Two independent reviewers screened titles, abstracts, and full-text articles for eligibility. Disagreements were resolved by a third reviewer. The PRISMA flow diagram (Figure 1) summarizes the study selection process.

### 2.6 Data extraction

Data were independently extracted using a predefined data collection form. Extracted data included: Study characteristics (authors, publication year, study design, country, number of centers), population demographics (sample size, age, sex distribution, surgical details), intervention characteristics (timing and technique of MMAE), and outcomes (recurrence, reoperation).

### 2.7 Risk of bias and quality assessment

The Cochrane Risk of Bias 2.0 tool was used to assess the methodological quality of included studies (17). Discrepancies in risk of bias ratings were resolved through consensus. The Grading of Recommendations Assessment, Development, and Evaluation (GRADE) approach was used to evaluate the certainty of evidence (18).

### 2.8 Statistical analysis

Data were analyzed using Review Manager (RevMan, Version 5.4). Odds ratios (ORs) with 95% confidence intervals (CIs) were calculated for dichotomous outcomes using a random-effects model. To assess statistical heterogeneity and inconsistency, the χ^2^ test and I^2^ statistic were utilized, with an I^2^ value of 50% or higher indicating substantial heterogeneity. The relative weight of each study, determined by its sample size, was taken into account when estimating the treatment effects.

## 3 Results

### 3.1 Search results and included studies

The PRISMA flow diagram details the systematic review process. A total of 1,804 records were identified through PubMed (421), Cochrane Library (23), and Google Scholar (1,360), with 351 duplicates removed. After screening titles and abstracts, 1,699 records were excluded. Of 105 full-text articles assessed, 103 were excluded for insufficient data or non-eligibility. Five RCTs were identified. The RCT by Fiorella et al. (12) was excluded because no recurrence or reoperation data stratified for surgically treated patients with or without MMAE were reported. The RCTs by Debs et al. (13) and Lam et al. (14) were excluded because bilateral SDHs were included in their study cohorts. Ultimately, 2 studies by Liu et al. (10) and Davies et al. (11) met the inclusion criteria for the meta-analysis. Figure 1 illustrates the process.

The EMBOLISE and MAGIC-MT trials evaluated middle meningeal artery embolization (MMAE) for chronic and subacute subdural hematoma (cSDH) (10, 11). Both studies excluded patients with bilateral subdural hematomas, focusing solely on unilateral symptomatic cases.

The EMBOLISE trial (11) conducted in multiple centers, included patients aged 18–90 years with symptomatic subacute or chronic SDH requiring surgical evacuation. Exclusion criteria included life expectancy <1 year, pre-existing severe neurologic impairment (Markwalder score ≥3), and significant disability (mRS ≥4). Patients were randomized 1:1 to receive surgery with adjunctive MMAE using the Onyx embolic system or surgery alone. MMAE was performed within 48 hours of randomization and targeted hematoma recurrence requiring reoperation as the primary endpoint. Fifty-three-point five percent of patients receiving adjunctive MMAE underwent burr-hole surgery, while 46.4% underwent craniotomy. Among those treated with surgery alone, 51.0% underwent burr-hole evacuation, and 49.0% underwent craniotomy. Subdural drains were used in 95.9% of patients receiving MMAE and 96.0% of those without MMAE.

The MAGIC-MT trial (10), a multicenter study in China, enrolled patients aged ≥18 years with symptomatic nonacute SDH and mass effect. Exclusions included bilateral SDH, craniotomy requirements, or emergency hematoma evacuation. In the EMBOLISE trial (11), 53.6% vs. 51.0% of patients in the treatment and control arms, respectively, were treated via burr-hole and 46.4% vs. 49.0% via craniotomy, whereas every patient in the MAGIC-MT (10) study underwent burr-hole evacuation. Subdural drains were inserted in 95.9% of EMBOLISE cases, while MAGIC-MT (10) only mandated drainage in the protocol without reporting the proportion actually placed. Participants were randomized to receive MMAE with Squid embolic agent followed by conservative management or burr-hole drainage, or usual care without MMAE. Primary endpoints were recurrence or progression of SDH within 90 days. MMAE was performed preoperatively in surgical patients. Patients who underwent craniotomy were excluded and the use of subdural drains was mandated in all cases. Table 1 summarizes the characteristics of the included studies.

**Table 1:** Study characteristics.

Characteristic	Davies et al., 2024 (11)	Liu et al., 2024 (10)
Design	Multicenter, open-label RCT	Multicenter, open-label RCT
Study sites	20 sites (USA)	31 sites (China)
Population	Adults 18–90 years with symptomatic subacute or chronic SDH requiring surgical evacuation	Adults ≥18 years with symptomatic nonacute SDH (subacute/chronic) causing mass effect
Exclusion	Bilateral SDH, severe comorbidities, pre-randomization mRS ≥4	Bilateral SDH, emergency surgery, life expectancy <1 year, or anatomical variations preventing MMAE
Interventions	Surgery ± adjunctive MMAE with Onyx	Surgery/conservative treatment ± adjunctive MMAE with liquid embolic material (Onyx)
Surgical approach	Burr-hole: 53.6% of the patients in the treatment group & 51.0% in the control group Craniotomy: 46.4% in the treatment group & 49.0%. in the control group	All patients underwent burr-hole surgery
Drains	95.9% received a surgical drain	Scheduled in protocol but no data regarding final drain placement
Primary endpoint	Recurrence/progression requiring reoperation within 90 days	Recurrence/progression of SDH within 90 days
Secondary endpoints	Functional outcomes (mRS), safety, adverse events	Hematoma thickness/volume changes, mRS outcomes, quality of life, adverse events
Sample size	400 patients	722 patients (total) 565 patients (surgically treated via burr-hole)
MMAE timing	Performed within 48 hours of surgery (before or after)	Performed before surgery in 99.6%

### 3.2 Risk of bias assessment

**Figure 2:**
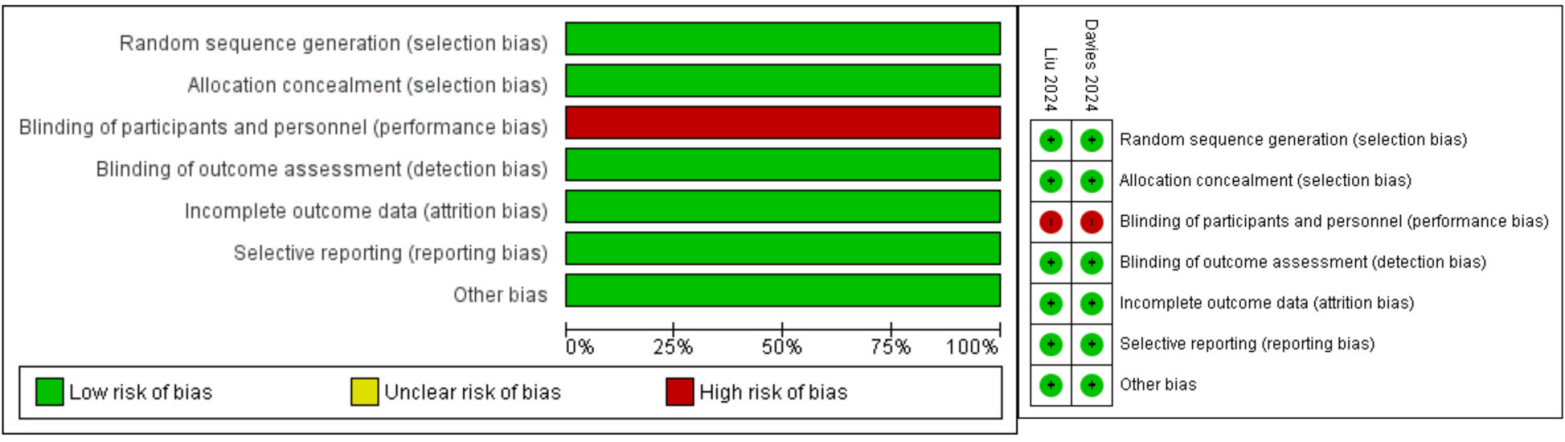
Risk of bias assessment for each kind of bias and summary of risk of bias of the included randomized controlled trials (reviewers’ judgments about each risk of bias characteristic of the included trials: “+” constitutes low risk; “−” constitutes high risk).

The risk of bias assessment for the two RCTs shows overall low risk of bias across most domains. Both studies demonstrated robust random sequence generation, allocation concealment, and complete outcome data reporting. However, performance bias was identified due to the lack of blinding of participants and personnel in both trials. Despite this, the blinding of outcome assessment and selective reporting were adequately addressed, ensuring high methodological quality. Figure 2 summarizes the risk of bias assessment.

### 3.3 Recurrence

**Figure 3:**
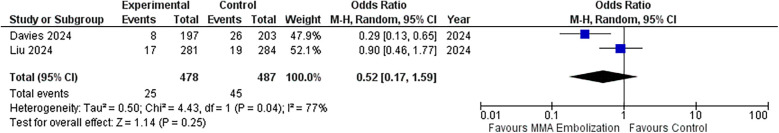
Forest plots displaying OR and 95% CI estimates for recurrence in studies evaluating MMA embolization compared to control in surgically treated SDH patients. Squares represent the odds ratio; the bigger the square, the greater the weight given because of the narrower 95% CI. Diamond represents the odds ratio of the overall data.

The meta-analysis included data from two randomized controlled trials (RCTs), comprising a total of 965 patients. Four hundred and seventy-eight (478/965; 49.5%) were allocated to the MMAE group, and 487 (487/965; 50.5%) to the control group. Twenty-five (25/478; 5.2%) in the MMA group had a recurrence, whereas 45 (45/487; 9.2%) in the control group had a recurrence. The odds ratio (OR) for recurrence or progression at 90 days was 0.52 (95% CI: 0.17–1.59; *p* = 0.25), indicating no statistically significant difference between MMAE and control groups. Heterogeneity was moderate (I^2^ = 77%, *p* = 0.04), suggesting variability between the included studies. Figure 3 illustrates the forest plot summarizing the results.

### 3.4 Reoperation

**Figure 4:**
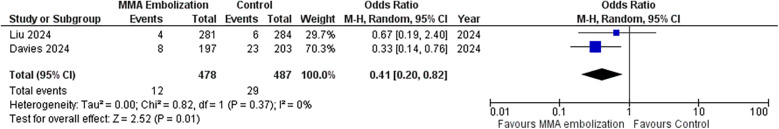
Forest plots displaying OR and 95% CI estimates for reoperation in studies evaluating MMA embolization compared to control in surgically treated SDH patients. Squares represent the odds ratio; the bigger the square, the greater the weight given because of the narrower 95% CI. Diamond represents the odds ratio of the overall data.

This section presents the results of a meta-analysis evaluating the impact of MMAE on recurrence or progression requiring reoperation at 90 days after surgical evacuation of unilateral cSDH. The meta-analysis included the data from the two randomized controlled trials (RCTs), comprising a total of 965 patients. Four-hundred and seventy-eight (478/965; 49.5%) were allocated to the MMAE group, and 487 (487/965; 50.5%) to the control group. Twelve (12/478; 2.5%) in the MMA group underwent reoperation, and 29 (29/487; 6.0%) in the control group. The OR for recurrence or progression requiring reoperation at 90 days was 0.41 (95% CI: 0.20–0.82; *p* = 0.01), indicating statistically significant reduction in recurrence with MMAE. Heterogeneity was low (I^2^ = 0%, *p* = 0.37), suggesting no variability between the included studies. Figure 4 illustrates the forest plot summarizing the results.

### 3.5 Publication bias

**Figure 5:**
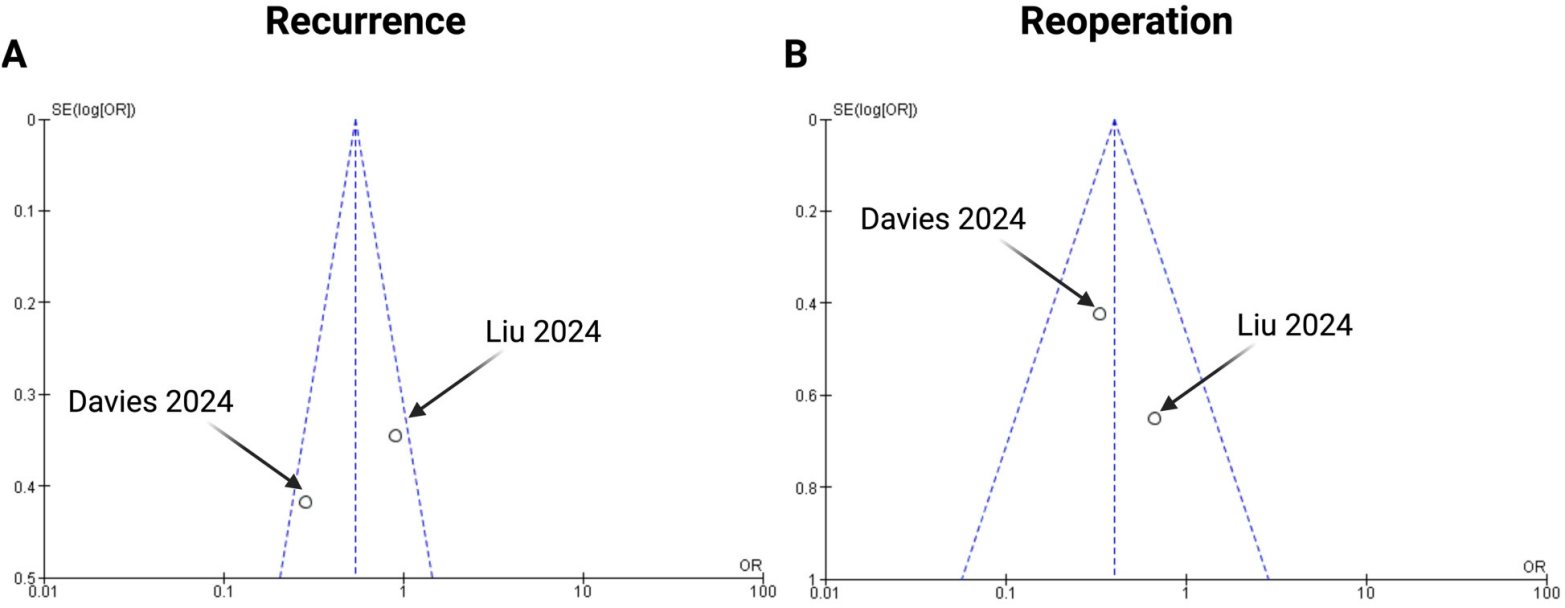
Funnel plots for the following endpoints of the present meta-analysis: **(A)** recurrence and **(B)** reoperation.

To ensure adequate reliability, we implemented three measures to assess potential publication bias. First, an extensive literature search strategy was employed. Second, the trials included in this meta-analysis strictly adhered to the predefined inclusion and exclusion criteria. Lastly, publication bias was analyzed using funnel plots (Figures 5) and statistical tests for key endpoints (recurrence and reoperation). The data points fell within the inverted funnel, suggesting minimal publication bias in the analysis of these endpoints.

### 3.6 Certainty of evidence

The GRADE assessment demonstrates high certainty for reduced reoperation risk (relative risk: 0.41, absolute reduction: 34 fewer per 1,000 patients), indicating a significant clinical benefit of middle meningeal artery embolization. However, moderate certainty supports recurrence reduction due to notable heterogeneity (I^2^ = 77%) and wide confidence intervals (OR: 0.52, CI: 0.17–1.59). Both outcomes are deemed critical for clinical decision-making. Supplementary Table 2 summarizes the GRADE approach.

## 4 Discussion

The present meta-analysis synthesizes data on the adjunctive role of MMAE in patients requiring surgical evacuation for unilateral subacute or chronic SDH. Our pooled analysis of 965 patients demonstrates a significant reduction in reoperation rates in patients receiving MMAE alongside standard surgical evacuation compared to surgery alone (see Supplementary Figure S2). These findings align with randomized controlled trials such as EMBOLISE and the pooling with the add of the surgical subgroup of MAGIC-MT strengthens the impact of MMAE as adjunctive to surgical evacuation of unilateral SDH (10, 11).

Consistent with recent studies, this analysis underscores the potential of MMAE to mitigate the recurrence associated with cSDH. For instance, the EMBOLISE trial reported a significantly lower risk of reoperation within 90 days among the treatment group compared to controls (4.1% vs. 11.3%; *P* = 0.008) (11). Similarly, the MAGIC-MT trial found a modest reduction in recurrence rates (6.7% in the embolization group vs. 9.9% in the control group), alongside a lower incidence of serious adverse events (10). In contrast, smaller-scale studies such as those by Lam et al. (14) investigating MMAE in both uni- and bilateral SDH patients failed to achieve statistical significance in recurrence reduction, primarily due to limited sample sizes. The present meta-analysis mitigates these limitations by integrating findings across trials, thus providing a broader perspective on the efficacy of MMAE.

Despite its advantages, the integration of MMAE into standard practice has faced challenges. Variability in the technical aspects of embolization, such as the type of embolic material used and procedural timing, has been reported. Furthermore, as noted in the literature, present RCT data on MMAE does not address other contributors to recurrence, such as inadequate hematoma evacuation (e.g., amount of irrigation, temperature of irrigation) or suboptimal postoperative management (e.g., drain time) (10–12, 19–21).

The randomized clinical trial EMPROTECT was published in June 2025 and adds relevant context to our findings (22). This multicenter trial randomized 342 patients who underwent surgery for a first or recurrent CSDH and were at high risk of recurrence to receive adjunctive MMAE or standard medical care. Although the primary endpoint—6-month recurrence—did not reach statistical significance (14.8% vs. 21.0%, adjusted OR 0.64; 95% CI 0.36–1.14; *p* = 0.13), the direction of the effect supports our findings and aligns with the risk reduction observed in our pooled 90-day data. Importantly, EMPROTECT reported a low complication rate and provides valuable evidence regarding the long-term safety and feasibility of MMAE in surgical patients. Taken together with EMBOLISE and MAGIC-MT, this emerging body of randomized evidence reinforces the potential role of adjunctive MMAE in reducing recurrence and reoperation rates in carefully selected cSDH patients, though further stratification by surgical technique and patient risk profile remains warranted.

## 5 Limitations and unaddressed variables

One critical limitation of the included studies, and consequently this meta-analysis, is the inability to analyze perioperative details (e.g., dexamethasone intake) or surgical details such as the volume of irrigation used, drainage duration, or variations in intraoperative technique. Trials like DRAIN TIME 2 and FINISH have shown that these factors significantly influence recurrence rates, with prolonged drainage and the use of irrigation being associated with improved outcomes (19, 21). Furthermore, in the EMBOLISE trial patient underwent either craniotomy or burr-hole surgery. The absence of standardized reporting on these surgical variables (craniotomy or burr-hole, drain time, amount and temperature of irrigation) restricts our ability to control for their effects in pooled analyses. Deeper investigations of the effect of MMAE in subgroups, such as stratification by surgical technique (burr-hole vs. craniotomy) or embolization protocol (timing, embolic agent), were not feasible due to the limited availability of detailed perioperative data in the included trials. Hence, this heterogeneity or unavailability of data regarding surgical approach, amount of irrigation, draining and medication intake (e.g., dexamethasone) limits the generalizability of the present evidence. Furthermore, data regarding functional outcome cannot be pooled among the present trials due to different applied scaling and reporting of outcome.

Additionally, patient heterogeneity in terms of age, comorbidities, and anticoagulation status further complicates interpretation. Future trials should aim to stratify SDH outcomes based on surgical parameters and patient characteristics to refine the indications for MMAE. These procedural differences may introduce heterogeneity in outcomes and limit the generalizability of the present evidence on MMAE. Ultimately, timing and frequency of follow-up imaging after surgical evacuation of chronic SDH is not standardized (23). This may lead to differing incidences of “recurrence”, and in turn of “reoperations”. These limitations underscore the need for future trials to implement standardized perioperative data collection and harmonized outcome reporting regarding surgical details to enable more robust analyses considering all relevant aspects of surgical SDH treatment.

## 6 Conclusion

In conclusion, the findings of this pooled analysis from 965 randomized patients suggest that MMAE, as an adjunct to surgery, could be considered for patients with unilateral symptomatic subacute or chronic SDH. Rigorous standardization of surgical and embolization protocols in future research will be pivotal in enhancing outcome predictability and patient safety.

## Data Availability

The original contributions presented in the study are included in the article/Supplementary Material, further inquiries can be directed to the corresponding author.
