# Supplementary material for: Adjunctive role of middle meningeal artery embolization in patients with surgical treatment of unilateral chronic subdural hematoma: a systematic review and meta-analysis of randomized controlled trials
Source: Front Surg. 2025 Jul 25;12:1623619. doi: 10.3389/fsurg.2025.1623619 (PMC12331645; doi:10.3389/fsurg.2025.1623619)
Supplement: Supplementary file 1 [file Table1.docx]

| **Supplementary table 1: Search syntax** | | | |
| --- | --- | --- | --- |
| **Database** | **Search String** | **Filters Applied** | **Results** |
| **PubMed** | ("subdural hematoma, chronic" OR "chronic subdural hematoma" OR cSDH OR "Hematoma, Subdural, Chronic"[Mesh]) AND ("Endovascular Procedures"[Mesh] OR “Intervention” OR "Embolization, Therapeutic"[Mesh] OR "angioembolization" OR "embolization" OR "embolization, therapeutic"[Mesh] OR "Meningeal Arteries"[Mesh]) AND ("surgical procedures, operative"[Mesh] OR "surgery" OR "surgical intervention" OR “Surgical evacuation” OR "Craniotomy"[Mesh] OR "burr hole drainage") AND ("recurrence"[Mesh] OR "recurrence rate" OR "recurrent chronic subdural hematoma") OR “progression” AND ("patient outcome assessment"[Mesh] OR "patient outcomes" OR "functional recovery" OR "neurologic status" OR “neurologic functioning” OR "quality of life" OR "complications"[Mesh] OR "adverse events" OR "treatment complications"[Mesh]) | None | 421 |
| **Cochrane library** | \| ("subdural hematoma, chronic" OR "chronic subdural hematoma" OR cSDH) AND ("Endovascular Procedures" OR "Embolization, Therapeutic" OR "angioembolization" OR "embolization" OR "Meningeal Arteries") AND ("surgical procedures, operative" OR "surgery" OR "surgical intervention" OR "Craniotomy" OR "burr hole drainage") AND ("recurrence" OR "recurrence rate" OR "recurrent chronic subdural hematoma") AND ("patient outcomes" OR "functional recovery" OR "neurologic status" OR "complications" OR "adverse events") \| \| --- \| | None | **23** |
| **Google Scholar** | "chronic subdural hematoma" AND ("Embolization" OR "Angioembolization" OR "Meningeal Arteries") AND ("surgical procedures, operative" OR "surgical intervention" OR "Craniotomy" OR "burr hole drainage") AND ("recurrence" OR "recurrence rate" OR "patient outcomes" OR "functional recovery" OR "complications") | Year: 2010–2024, Publish or Perish Tool | **1360** |
