# Supplementary material for: Adjunctive role of middle meningeal artery embolization in patients with surgical treatment of unilateral chronic subdural hematoma: a systematic review and meta-analysis of randomized controlled trials
Source: Front Surg. 2025 Jul 25;12:1623619. doi: 10.3389/fsurg.2025.1623619 (PMC12331645; doi:10.3389/fsurg.2025.1623619)
Supplement: Supplementary file 2 [file Table2.docx]

**Supplementary Table 2: Certainty of evidence according to GRADE approach for prehabilitation versus rehabilitation**

| **Certainty assessment** | | | | | | | **№ of patients** | | **Effect** | | **Certainty** | **Importance** |
| --- | --- | --- | --- | --- | --- | --- | --- | --- | --- | --- | --- | --- |
| **№ of studies** | **Study design** | **Risk of bias** | **Inconsistency** | **Indirectness** | **Imprecision** | **Other considerations** | **MMA** | **Control** | **Relative (95% CI)** | **Absolute (95% CI)** |  |  |
| **Reoperation** | | | | | | | | | | | | |
| 2 | randomised trials | not serious | Low | Low | Low | none | 12/478 (2.5%) | 29/487 (6.0%) | **0.41 (0.20-0.82)** | **34 fewer per 1000** | ⨁⨁⨁⨁ High | CRITICAL |
| **Recurrence** | | | | | | | | | | | | |
| 2 | randomised trials | not serious | High^a^ | Low | Low | none | 25/478 (5.2%) | 45/487 (9.2%) | 0.52 (0.17-1.59) | **40 fewer per 1000** | ⨁⨁⨁◯ Moderate | CRITICAL |

CI: confidence interval; OR: odds ratio; a. The inconsistency index was 77%
